# Improving Debt Literacy by 2/3 Through Four Simple Infographics Requires Numeracy and Not Focusing on Negatives of Debt

**DOI:** 10.3389/fpsyg.2021.621312

**Published:** 2021-03-26

**Authors:** Robert Porzak, Andrzej Cwynar, Wiktor Cwynar

**Affiliations:** ^1^Experimental Psychology Lab, Faculty of Human Sciences, University of Economics and Innovation, Lublin, Poland; ^2^Institute of Public Administration and Business, University of Economics and Innovation, Lublin, Poland

**Keywords:** debt education, visual attention, numeracy, graph literacy, linguistic literacy, focus on debt positives and negatives, debt literacy

## Abstract

Borrowing behavior may be more resistant to formal educational treatments than other financial behaviors. In order to study the process and results of infographics-based debt education, we used eye tracking technology (SMI RED 500 Hz) to monitor the oculomotor behavior of 108 participants (68 females) aged 18 to 60 who were shown 4 infographics. The study used an experimental design with repeated measures and an internal comparison group. We also used scales of debt literacy and a set of information literacy scales: numerical, graph, and linguistic. The results confirm that short-term infographics-based debt education can improve debt literacy significantly. The difference in processing the educational contents that were not known to participants before the educational session suggests that participants with better information literacy make more considerable debt literacy progress. Specifically, we found that numerical literacy is a significant mediator of debt education results, depending on the initial level of debt literacy; this relation is moderated by the focus of visual attention on negatives of debt. We found no significant relationship between debt literacy education results and those of graph and linguistic literacy.

## Introduction

Financial decisions are unavoidable, at least in adulthood. As in any other domain of life, the ability to make the right decision depends on the extent to which the individual possesses appropriate knowledge and skills—something known as a core of “financial literacy” (Huston, 2010; Remund, 2010). Financial knowledge and skills pertain to various aspects of household finance, such as cash management, savings, investment, retirement preparation, insurance, and borrowing (Lusardi and Mitchell, 2007, 2011a; Yoong, 2010; Alessie et al., 2011; Lusardi and Tufano, 2015). Overall, these aspects can be broadly divided into those that are linked to the asset side of household budgets and those linked to the liabilities side. The asset side requires households to address only two basic questions: *how much* and *how* to save or invest to achieve financial security and long-term well-being. The question of *whether* to save or invest is, thus, rhetorical in this case.

However, the question is not rhetorical on the liabilities side of household budgets. In contrast to saving or investing decisions, a wrong borrowing decision may lead to a literal financial default, that is, the inability to repay debt. As a result, consumers' attitudes toward debt (loans) are more likely to be negative than positive (Almenberg et al., 2018; Białowolski et al., 2020). It has also been evidenced that borrowing behavior may be more resistant to formal educational treatments than other financial behaviors (Miller et al., 2015; Kaiser and Menkhoff, 2017). Coupled with debt literacy being lower than financial literacy within a specific population (van Ooijen and van Rooij, 2016), this may suggest that the acquisition of debt literacy is more difficult than that of literacy in other financial domains; however, some studies suggest that debt-related education gives better results in terms of behavior than education regarding savings (Robinson et al., 2016). In our study, we extend previous findings in the area of financial education with regard to debt literacy and debt decisions. More precisely, we focus on the scarcely studied effectiveness of visual educational techniques (Heinberg et al., 2014) and test whether visual attention (measured with eye tracking) affects debt literacy education. To the best of our knowledge, this is the first study that aims to discover not only the effectiveness of financial education, but also its underlying process.

Debt literacy is an important domain-specific aspect of financial literacy. It is defined as the ability to make simple, everyday decisions regarding debt contracts (Lusardi and Tufano, 2015). Initial studies revealed a low level of debt literacy in the population: many consumers do not understand how credit cards work and others do not comprehend interest compounding (Disney and Gathergood, 2011; Lusardi and Tufano, 2015; van Ooijen and van Rooij, 2016; Cwynar et al., 2018a). This has an adverse effect on financial behavior and outcomes. People with low financial competence tend to have costly mortgages, are less prone to refinance their mortgages when interest rates are falling, and have a tendency to engage in high-cost transactions (i.e., choose high-cost borrowing or pay higher fees) (Moore, 2003; Campbell, 2006; Lusardi and Tufano, 2015). Moreover, people with low debt literacy report excessive debt loads or an inability to evaluate their debt position (Lusardi and Tufano, 2015). Thus, it is not surprising that debt is perceived as a stressful burden resulting in a sense of uncertainty (The Handlowy Leopold Kronenberg Foundation, 2014) and leading to serious physical and psychological disorders (Fitch et al., 2007; Jenkins et al., 2008; Archuleta et al., 2013; Sweet et al., 2013; Amit et al., 2020).

Given the low levels of debt literacy in the population, it seems reasonable to design interventions that can compensate for these shortcomings in debt literacy. Despite some controversies around the effectiveness of financial education (Willis, 2011; Fernandes et al., 2014), we believe that one of the most promising ways to help people avoid incorrect decisions that are partly to the result of a lack of financial competence is to educate them and enhance their financial and debt literacy. Generally, previous studies have revealed that financial education shapes financial literacy, which, in turn, may lead to better (more healthy) financial decisions, as reflected in the most recent meta-analyses (Miller et al., 2015; Kaiser and Menkhoff, 2017, 2018).

The aim of our study is twofold. First, we examine whether and how infographics-based education affects debt literacy levels. Our intention is to test whether financial micro-education is effective, and the conditions under which this happens. However, financial educational interventions operate like a black box: we know the input (the content and form of such education) and output (financial literacy and behavior), but we do not know their internal mechanism. Therefore, we designed our study to go one step further and test the processes underlying effective education in the field of debt education. In our study, we decided to focus on visual educational techniques because they are likely to be particularly efficient (Mirel et al., 2016; de Haan et al., 2018); they enable easy access, do not require a major time commitment, and meet the civilizational and cultural changes reflected in image-based communication and the shift of human activity to the online sphere. Moreover, the media and Internet are among the top sources for learning about financial management, especially credit practices (Hilgert et al., 2003). People who report learning about finance from the media or the Internet are significantly more effective at credit management than people who do not report learning from these sources.

The second aim of our study is to test whether visual attention focused on positive or negative aspects of debt, numerical literacy, graph literacy, and linguistic literacy moderates the effectiveness of financial education. Previous studies have shown that these factors may play an important role in learning effectiveness and in financial literacy acquisition (Banks and Oldfield, 2007; Okan et al., 2016; Hoffmann and McNair, 2019).

## Literature Review

### Financial Education and Its Effectiveness

The literature on the effectiveness of financial education in formal setting is extensive. Despite the inconclusive results emerging from reviews of research in this area (Hathaway and Khatiwada, 2008; Collins and O'Rourke, 2010; Gale and Levine, 2010; Fernandes et al., 2014), recent meta-analyses provide evidence that financial education is overall effective, although its effects vary widely across programs (Miller et al., 2015; Kaiser and Menkhoff, 2017, 2019). This variation may be attributed to a number of reasons. First, the effectiveness of financial education may be contingent on its content. Second, the effectiveness may depend on the transfer mechanism (form of education). Third, both the content and the form can be effective (or, conversely, ineffective) but the downstream evaluation process is unable to efficiently capture the effects of the intervention. Brown et al. (2016) confirmed that, indeed, the impact of financial education on debt behavior of young people depends on the educational content: numerical and strictly financial content has a positive influence on this behavior, and more general economic content leads to worse behavior. In turn, Brugiavini et al. (2018) showed that most financial education programs do not involve any subsequent evaluation.

The form of educational treatments has been investigated to a limited extent. Carpena and Zia (2011) point out that the literature focuses on the end outcomes of financial education, i.e., whether and in what direction this education alters behavior. Much less is known about the process of the change, i.e., why individuals do (or do not) increase their financial literacy and modify their behavior in response to an educational program and how the change comes about. A scrutiny of program design and delivery—i.e., the form of the program—opens up new avenues for exploring both the morphology and the dynamics of the change. This very issue—how consumers acquire financial literacy in formal educational setting—is pivotal to our article. Visual educational techniques, especially those used online, are particularly understudied forms of educational program design and delivery (Heinberg et al., 2014).

The literature on the impact of financial education on debt literacy and behavior is sparse. Researchers mostly focus on financial management in general or on retirement-related issues. However, it has been established that education aimed at promoting healthy credit management behavior may face particular challenges. Miller et al. (2015) found that unlike in the case of household bookkeeping and saving behavior, where financial education had a positive impact, it did not entail improvements in terms of loan repayment. A similar result has been reported by Kaiser and Menkhoff (2017), who state that handling debt is more difficult to influence through financial education. Miller et al. (2015) argues that this is the case because debt-related behavior is significantly more influenced by external and uncontrollable factors compared to behavior in other subdomains of household financial management. For example, an income situation that makes it difficult to make ends meet, or unexpected and costly health problems or job loss, may encourage people to go into debt or delay repayment despite knowing the consequences. Perhaps the findings on the role of financial education in shaping debt behavior differ from those concerning behavior in other subdomains of financial management also because the diversity of credits and loans has not been sufficiently investigated to date in the educational context. For instance, the findings of Wagner and Walstad (2019) suggest that financial education may have a larger impact on long-term and non-recurring behavior that does not provide frequent feedback (e.g., mortgage decisions), and a weaker impact on short-term behavior, which, due to timely feedback, is mostly shaped through learning-by-doing (e.g., credit card usage).

The literature on the impact of financial literacy on credit choices and debt management behavior is more extensive. Several studies revealed that more financially literate consumers are more likely to be holders of secured debt, mostly mortgages (Disney and Gathergood, 2011; Brown and Graf, 2013; Feng et al., 2019; Bialowolski et al., 2020). Bialowolski et al. (2020) provides arguments that mortgage borrowing can be considered healthy financial behavior. At the same time, the correlation between financial literacy and the likelihood of holding unsecured (i.e., riskier and more costly) debt is absent (Feng et al., 2019) or negative (Brown and Graf, 2013). Higher financial literacy scores are associated with higher debt loads in both conditional (Feng et al., 2019) and unconditional comparisons (Disney and Gathergood, 2011). However, despite having larger debt balances, borrowers with higher financial literacy incur lower debt costs (Disney and Gathergood, 2011). This suggests more sound and prudent borrowing behavior on the part of more financially literate consumers.

Several studies have shown that more financially literate mortgage borrowers report lower mortgage interest rates (Moore, 2003; Huston, 2012; Bialowolski et al., 2020). They have also been found to be less likely to reach out for exceptionally costly borrowing vehicles, such as payday loans, auto-title loans, mail order catalog debt, pawnshop debt, etc. (Chatterjee, 2013; Disney and Gathergood, 2013; Lusardi and de Bassa Scheresberg, 2013; Lusardi and Tufano, 2015; Robb et al., 2015). Fornero et al. (2011) established that those more financially literate more often shop around when they need a mortgage, while those less literate more often accept the first offer they receive. Higher levels of financial literacy are also positively associated with more accurate self-assessment of one's own mortgage contracts (Courchane et al., 2008) and a greater ability to better match loan products to one's own situation (Fornero et al., 2011; Smith et al., 2012; Gathergood and Weber, 2017). Finally, more financially literate debtors are less likely to default on their debts (Gerardi et al., 2010; Fornero et al., 2011; Agarwal et al., 2017).

### Forms of Financial Education and Specific Nature of Online Education

Researchers have so far rarely addressed the role of the form in which financial education programs are delivered, although the range of available forms of such education is very broad. These may be classes in the form of a lecture, a seminar, or a workshop. Such in-person classroom-style sessions can involve the use of equipment and software that make it possible to simulate real decision-making conditions, or the use of various types of games (e.g., board games or computer games). Another form is one based on interaction without direct expert participation (teacher, trainer, tutor), resembling typical e-learning courses—e.g., multimedia presentations, scripts with case studies, video or audio material, infographic material available online and many others.

While traditional print materials and in-person workshops are still the most common, advances in technology have created online financial education opportunities in recent years (Kim et al., 2017). This trend toward online education has been lately reinforced by the Covid-19 pandemic. In fact, online education is a response to the demands of modern times. On the one hand, many aspiring consumers do not have time for traditional further education. On the other hand, lifelong learning is becoming an integral part of our lives. In such a situation, online education becomes a natural option. However, it is important to remember that it has both advantages and disadvantages.

In terms of advantages, online financial education is an adequate answer to the need for learning “at teachable moments” (Miller et al., 2015; Kaiser and Menkhoff, 2017). All meta-analyses covering research on financial education effectiveness (Fernandes et al., 2014; Miller et al., 2015; Kaiser and Menkhoff, 2017) stress that the essential condition here is the ability of a program to intervene “just in time.” Online education meets this condition as it is easily accessible, anytime and anywhere, and does not require a major time commitment. Low cost of access makes the circle of recipients of online educational programs significantly wider compared to traditional ones. Kim et al. (2017) argues that online financial education should be approached in terms of principles of behavioral economics. They observe that online education (especially with the use of mobile devices) provides unique opportunities to impact behavior directly without overloading consumers with specialized knowledge (OECD, 2019). For instance, participation in an online financial educational course may involve receiving automatically app-generated prompts or reminders helping to overcome some negative behavioral habits. On the negative side is that not all online resources are reliable and they are not always developed according to pedagogical principles or with a sufficient consideration of the psychological conditions of the learning process, especially in the case of some groups of learners. Privacy and security also matter (Kim et al., 2017).

Hubbard et al. (2016) conducted a laboratory experiment in which 86 students were randomly assigned to three groups exposed to financial education content. The content was the same in all groups and concerned compound interest; in all cases it was delivered online. However, it was presented differently in each group: in textual form, in the form of traditional linear graphs, and in the form of volumetric graphs. Significant improvements in the understanding of the concept of compound interest were recorded in the groups exposed to text and volumetric graphs. There was no significant improvement among the students using linear graphs.

Heinberg et al. (2014) demonstrated financial content to participants of their field experiment in two different formats: short written narratives and equally short videos (in each case, ~3 min were required). The study showed that both the video and the more traditional descriptive material (narratives) had a significant positive effect on improving the score on a financial literacy test taken shortly after the exposure. It was also found that the positive impact largely persisted in the medium term: when measured again eight months after the educational intervention, between one-quarter and one-third of the gains in financial knowledge persisted.

Lusardi et al. (2017) expanded on Heinberg et al. (2014) study by comparing four forms of educational content delivery: a brochure, a visual interactive tool, a written narrative, and a video narrative. The study showed that all four formats significantly increased the levels of both financial self-efficacy and self-assessed knowledge compared to the control group. In financial knowledge test scores, an increase over the control group was noted only in the groups using video and brochure material. Collins and Urban (2016), in turn, studied the effectiveness of self-paced educational material that was available online. Their findings suggest that this formula for increasing financial knowledge can be effective, and that it can foster desired financial behaviors.

### Presumable Moderators of Financial Education Effectiveness

The visual attention process is understood as “a set of cognitive operations that mediate the selection of relevant and the filtering out of irrelevant information from cluttered visual scenes” (McMains and Kastner, 2009). The definition of visual attention as a top-down-driven “foveation of a stimulus” (Posner and Petersen, 1990; Petersen and Posner, 2012) during free viewing emphasizes the conscious processing of information. Definitions that present visual attention as a conscious top-down process may be most helpful in analyzing a debt education course. The visual attention properties of span, selectivity, and sustainability are positively correlated with education ability (Bosse and Valdois, 2009). Visual attention selectivity is one of the most fundamental cognitive functions, allowing humans and other primates to confine themselves largely to stimuli that are relevant to behavior (Moore and Zirnsak, 2017) and influence many cognitive and behavioral processes. The mode and results of solving verbal problems depend on the focus of visual attention on the stimuli: center-focused objects stimulate analytic problem solving, whereas broad-space-located objects stimulate the solving of verbal problems by using insight (Madsen et al., 2012; Wegbreit et al., 2012). The longer the visual attention lingers on information important for a consumer, the higher the probability is of positive buying decisions by consumers (Grebitus et al., 2015; Rihn and Yue, 2016).

Studies on visual perception indicate that top-down visual attention can be activated in parallel with bottom-up driven object recognition processes, afforded by bidirectional information flow (Noorman et al., 2018). Linguistic cues guide interpretation of visual scenes (top-down), while perceptual information shapes interpretation of linguistic input (Vulchanova et al., 2019) and acoustic neurofeedback might improve the process of mental rotation of 3D objects (bottom-up) (Ozga et al., 2019). The interaction between top-down and bottom-up driven visual and auditory attention again raises questions about the learning styles hypothesis. The learning styles idea understood as preferences for visual or auditory stimuli (Cuevas and Dawson, 2018), or preferences for processing pictures or words (Dunn and Price, 1980; Rayner and Riding, 1997; Thepsatitporn and Pichitpornchai, 2016), has poor research evidence to support and has been termed the “meshing hypothesis” (Coffield et al., 2004; Pashler et al., 2008; Rogowsky et al., 2015, 2020; Willingham et al., 2015). However, it is used in educational practice and favored by many academics (Newton and Miah, 2017).

The information describing the positives or negatives of debt can be understood as an expected reward or loss, moderating borrowing behavior. Monetary reward and punishment incentives activate motivational neural circuitry and increase its functional coupling with the cognitive control networks (Cubillo et al., 2019). Cloninger described reward dependence as one of the neurologically based personality dimensions, together with novelty seeking and harm avoidance (Cloninger, 1986). Reward and punishment activate the same areas of the brain—the medial orbitofrontal cortex—and amount to the same thing for the brain: achieving the goal (Gross, 2006). Tversky and Kahneman (Tversky and Kahneman, 1992) noticed that the loss aversion tendency directs human decisions under uncertainty. Attitudes toward risk determine the level of household debt (Brown et al., 2013). Risk preference increases the probability of taking risky debt decisions (Jiangqun and Xiaoyan, 2012; Wang et al., 2018). Avoiding the negative consequences of borrowing risks can protect from wrong debt decisions. We verify whether the focus on positives or negatives of borrowing can moderate the process of debt education.

Numerical literacy is defined as the ability to process basic probability and numerical concepts (Fagerlin et al., 2007; Peters et al., 2016). Numeracy has been studied many times in the financial context both as a component of financial literacy (French and McKillop, 2016; Bannier and Schwarz, 2018) and as a concept that is distinct from literacy (Banks and Oldfield, 2007; Cole et al., 2011; Roa et al., 2019). It is well established that numeracy has a profound impact on financial decisions. People with higher numeracy skills are much more likely to participate in the stock market (Lusardi and Mitchell, 2011b), are less likely to fail with their mortgage payments (Gerardi et al., 2010), and are less likely to report difficulties in paying off their debt (Disney and Gathergood, 2011). Thus, we hypothesize that numerical literacy might moderate the effectiveness of financial education, that is, people with higher numerical abilities might comprehend financial concepts more easily; this results in quicker and deeper educational effects in them than among people with low numerical abilities.

Graph literacy, on the other hand, is defined as the ability to understand the graphically presented information (Garcia-Retamero et al., 2016; Okan et al., 2016). Previous studies revealed that individuals generally differ significantly in their ability to understand graphical information and derive benefits from visual forms of information (Galesic and Garcia-Retamero, 2011). In other words, people with higher graph literacy are more accurate in their interpretations of the presented information (Shah and Freedman, 2011) thus, it seems reasonable to test whether the effectiveness of financial education designed visually is moderated by graph literacy.

Linguistic literacy can be viewed as a constituent of language knowledge and is characterized by the availability of multiple linguistic resources and the ability to consciously access one's own linguistic knowledge and view language from various perspectives (Ravid and Tolchinsky, 2002). Chomsky defined *linguistic performance* as the ability to produce and comprehend sentences in a language (Chomsky, 1965; Knowles, 2000). Because we did not find studies describing the role of linguistic literacy in financial education, we decided to test whether people with higher linguistic literacy learn debt-related information faster and with better results.

The set of information-processing literacies is treated in education and by librarians as a part of information literacy. Information literacy is defined as “the adoption of appropriate information behavior to obtain, through whatever channel or medium, information well fitted to information needs, together with critical awareness of the importance of wise and ethical use of information in society” (Webber and Johnston, 2017, p. 158). Increasing information literacy improves students' (Lawson and Brown, 2018) and teachers' (Saglam et al., 2017) critical thinking and selection of useful information. We assumed that information literacy consists of numerical, graph and linguistic literacy.

## Materials and Methods

Based on the literature review, which suggests that visual attention focused on the positives and negatives of borrowing, as well as numerical, graph, and linguistic literacy, could affect debt literacy education, we hypothesize that:

H1. Participants focusing visual attention on infographics longer show better debt literacy education results.

H2. Participants with higher numerical, graph, and linguistic literacy show better debt literacy education results.

Verification of these hypotheses was conducted in an experimental design with repeated measures and with an internal comparison group.

### Ethics Statement

This study was approved by the University of Economics and Innovation Ethics Committee. The study was carried out in accordance with relevant guidelines and was conducted according to the principles expressed in the Declaration of Helsinki. Before data collection, all participants were informed about the study protocol and gave their consent to take part in the study.

### Participants

Setting the alpha level at 0.05, and expecting a medium effect size (Cohen's d = 0.5), we assumed minimal statistical power *P* = 0.8 (Cohen, 1988) and participants' allocation to the control/experimental groups = 0.8. The total sample size calculated according to the assumptions above was *N* = 102 for one-tail *t*-tests and *N* = 108 for the one-tail Wilcoxon-Mann-Whitney tests. The minimal sample size for ANOVA with 4 repeated measures in the 2 groups is *N* = 22 (Faul et al., 2007). Adding 1/3 to the highest requested sample size (*N* = 108) due to the possible data loss resulted in minimal sample size *N* = 144. Finally, 176 persons participated in the experiment, which ensured meeting requirements for minimal statistical power of results.

The participants were invited to take part in the experiment *via* the electronic administration system among extramural and postgraduate students (adults, age ~30) of the University of Economics and Innovation in Lublin (Poland). The information provided to participants described the experiment as being focused on the assessment of the correlates of debt literacy and infographics stimuli that were best for educational purposes. The participants were informed about the use of eye-movement tracking. Participants could select one of the following incentives proposed: a financial fee, compensating proportionally time spent in the lab (median per hour salary in Poland ≈ PLN 20 ≈ USD 5) or up to 5% of the semester exam points. The compensation in both groups depended on the sum of points collected in the Debt Literacy tests, which were administered twice in the experiment. Almost 99% of students selected semester exam points and received course credit for participation.

Participants with significant vision defects, such as astigmatism, myopia of above 5 dioptres, or uncorrected myopia of an unknown scale were excluded from participation before the start of the experiment. Random dropouts also occurred during other stages of the data collection process. We recorded the drop-out of 38% of participants from the experimental procedure due to visual impairment and errors in completing the questionnaires. The eye tracker calibration for 27 participants did not meet the acceptable threshold. Their participation in the experimental procedure was accepted with no comments to avoid stress and frustration, and all data were collected. However, their results were excluded from all analyses. Eighteen participants omitted at least one answer in the questionnaires, resulting in a lack of data. The 23 participants who showed a lack of fixation on at least one of the presented infographics were excluded during the data processing that took place before the final calculations; however, compensation points were granted to all participants. Final analyses were performed on data collected from 108 participants (68 females and 40 males, aged 18 to 60). The mean age of the participants was 33.46 years (SD = 9.66). The sample is representative for the general population of working adults in Poland in terms of age. However, the gender gap [overrepresentation of women, typical for university education (Baker, 2016; Foley, 2019)] and the level of education (at least secondary) limit the generalization of results.

### Procedure

To avoid selection bias, participants were randomly assigned to an experimental (E) or a control (C) group of the ratio 5:4−60 participants to the experimental group and 48 to the control group. Due to the fact that individuals usually acquire information from left to right and from top to bottom (Ishii et al., 2011; Polonio et al., 2015) the position of the information referring to the negatives and positives of debt on each infographic (left- or right-hand side of the infographic) was additionally counterbalanced across subjects in a double-blind model. Finally, we extracted *ex post* two subgroups of participants of the experimental group using the change in the debt literacy score: those participants who increased their debt literacy were classified to the first group and those who had no increase in debt literacy were in the other group.

**Figure 1:**
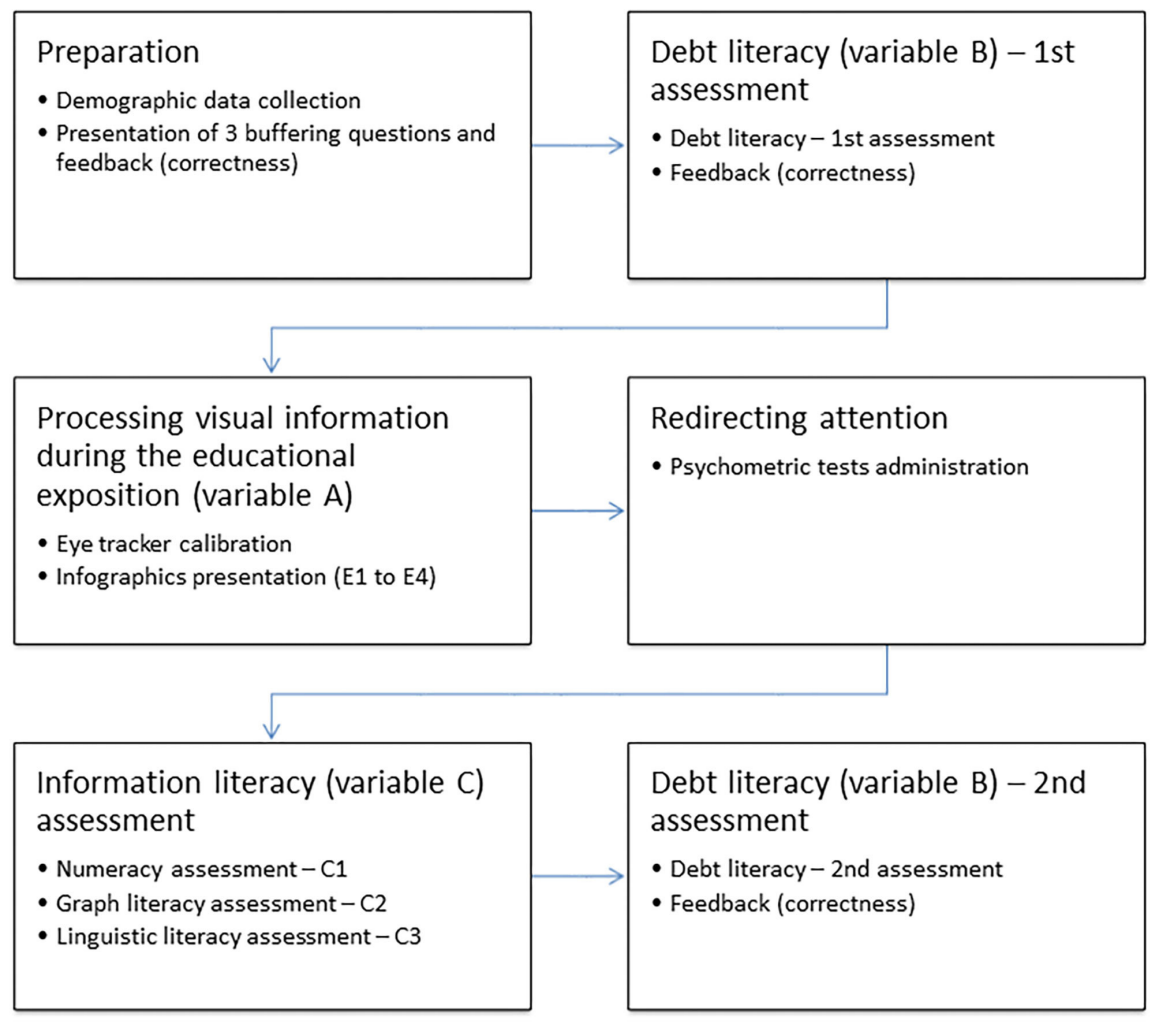
The experiment outline.

Figure 1 shows the experimental procedure. At the beginning of the experiment, demographic data was solicited (i.e., gender, age, and education). This was followed by short training on the non-visual use of the keyboard. Next, we asked participants to complete the Debt Literacy Scale for the first time in the experiment; this was followed by feedback on the results in terms of the percentage of correct answers. Then, we presented the sequence of educational infographics (infographics, henceforth) and the oculomotor activity was recorded during this task by using the eye tracker. The time spent on each infographic was not limited. Participants who had completed the debt education with infographics were asked to complete, in another room, a psychometric questionnaire not connected with the experiment, and additionally, the following scales designed to be used in the present study: the numeracy scale; graph literacy scale; and linguistic literacy scale. Completing all the questionnaires took about 1 h. The short time between measures on the one hand, and concentration on completing the questionnaires on the other, eliminated the maturation and general education bias and the possibility of exchanging information between students about their answers and results. At the end of the experiment, the debt literacy level was measured again in both groups in order to verify the role of infographics in debt education (the assumed debt literacy change in group E) and to control testing effects for the Debt Literacy Scale (the assumed lack of debt literacy change in group C).

### Measures

#### Debt Literacy

Debt literacy (variable B), defined after Lusardi and Tufano, as “the ability to make simple decisions regarding debt, applying basic knowledge about interest compounding to everyday financial choices,” (Lusardi and Tufano, 2015, p. 333) was measured with the Debt Literacy Scale comprising three questions; another question (Cwynar et al., 2019) was added to strengthen understanding of the process logic in debt education:

“You have taken a PLN 5,000 loan for one year and the interest rate you are charged is 10% per year. You are given the following two options to pay the interest on the loan:

- A one-time payment of PLN 500 in advance (at the beginning of the year), which means that PLN 4,500 will be effectively available to you on the day the loan is granted.
- A one-time payment of PLN 500 at the end of the year, which means that PLN 5,500 will have to be returned to the lender on the day of repayment.

Which is the more advantageous option?”

Each answer was assessed as correct (1) or incorrect (0), and points were allocated on a scale ranging from 0 to 4.

#### Numeracy, Graph Literacy, and Linguistic Literacy

Information literacy was limited in the current study to information presented numerically, graphically, and verbally (variable C). These three dimensions were assessed by using brief subjective assessment scales administered separately. Two standardized self-reported scales for numeracy and graph literacy were adapted, and a new scale for assessing linguistic literacy was applied. Numeracy was assessed using the eight-item subjective numeracy scale (Fagerlin et al., 2007; Zikmund-Fisher et al., 2007). Graph literacy was measured with the use of the five-item Subjective Graph Literacy Scale (Garcia-Retamero et al., 2016).

The Subjective Linguistic Literacy Scale developed by Robert Porzak is based on Chomsky's well-known *linguistic performance* concept (Chomsky, 1965; Knowles, 2000) and the definition of *linguistic literacy* (Ravid and Tolchinsky, 2002). Statements were formulated on the basis of Common European Framework of Reference for Languages standards (Common European Framework of Reference for Languages, 2001). The Subjective Linguistic Literacy Scale consists of five questions asking respondents to assess their linguistic ability in different contexts:

1. How good are you at understanding journalistic messages from TV or radio?
2. How good are you at understanding long articles read in popular newspapers?
3. How good are you at differentiating in conversation shades of meaning of your statements?
4. How good are you at formulating logical statements in a style appropriate for a given context?
5. How good are you at describing complex issues in mails, studies or articles?

Participants responded to subjective linguistic literacy questions by selecting one of the six options ranging from “1 = Not at all good” to “6 = Extremely good.”

### Research Instruments

The presentation of stimuli and measurement of visual attention on negatives and positives of debt as presented in the infographics was carried out on a 21″ LCD screen (1920 x 1080 px) using E-Prime 2.0 software (Psychology Software Tools, Inc.). The behavioral responses were collected using a standard computer USB keyboard. An SMI RED500 eye tracker at a sampling frequency of 500 Hz was used for the registration of eye movements and fixations in areas of interest (AOI). The spatial accuracy of RED500 was 0.4 degrees. Calibration accuracy was kept below 1° of visual angle, which ensured high time resolution and spatial precision of fovea location.

#### Stimuli

An ecological protocol exploited stimuli selected from the debt educational campaign as a sequence of infographics reflecting each aspect of debt literacy described in the Debt Literacy Scale. Each stimulus was prepared as an RGB bitmap of 1920 x 1080 px containing two infographics: the first one informing about the positive aspects of debt (pluses, opportunities), and the other one informing about the negatives (minuses, risks). The size of each infographic was set to a width of 500 px and a height of 400 px, which subtended 11.2° x 9.0° of visual angle in size, with 268 px (6°) separation between infographics.

**Figure 2:**
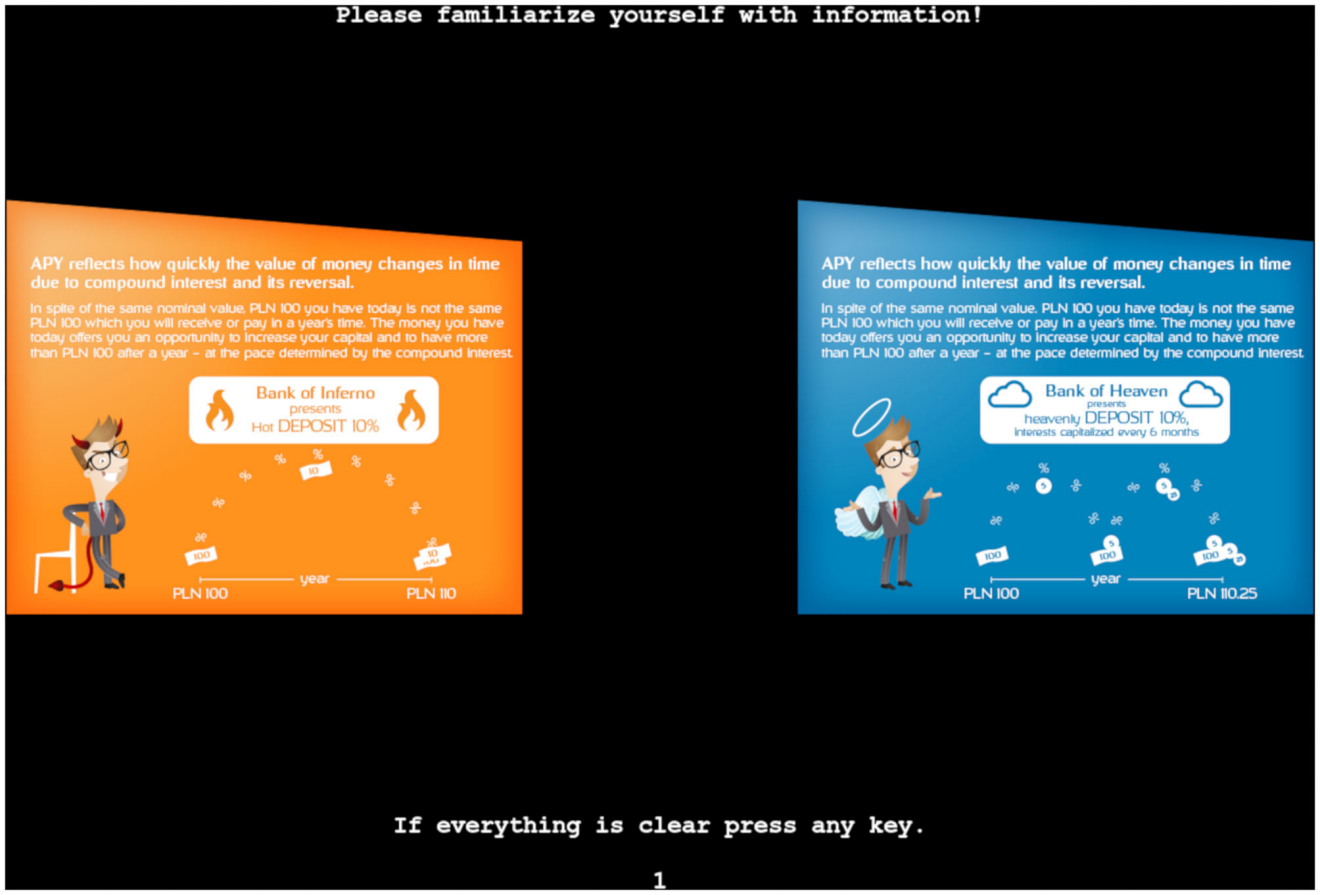
Representative infographic (1)—Negatives vs. positives of calculating and not calculating compound interest.

Each stimulus had two versions: (1) an infographic that had the negatives on the left-hand side of the screen and positives on the right-hand side; and (2) another infographic with the positives on the left and negative on the right. The presentation of stimuli versions was counterbalanced in the groups of participants so that an equal number of stimuli were assessed with the positives located on the left and right. Figure 2 shows a version of one of the infographics used.

The infographics were arranged sequentially, representing the increasing complexity of the properties of loans [as reflected in the results of debt literacy surveys; see, for instance (Lusardi and Mitchell, 2011a; Cwynar et al., 2018a), that is, calculating compound interest, calculating interest rate, comparing payment frequency, and comparing payment dates. The top part of each infographic described debt properties verbally and was the same for the positive and negative aspects; the differences were highlighted by the use of graphical elements. The stimuli for the control group were exactly the same in shape, structure, and color, but contained general and meaningless information not connected directly with debt or economic education; it had “economics-related” words, such as “economy,” “value,” and so on. The full set of infographics with original and translated versions can be found on the online repository at http://dx.doi.org/10.17632/f5s6cxk38f.1.

### Behavioral Analyses

The increase in the score obtained for the answers to the Debt Literacy Scale was analyzed to check for debt literacy. The difference between the Debt Literacy Scale results assessed before the presentation of infographics and one hour after the presentation was an indicator of short-term infographics-based debt literacy education results.

The mean dwell time (defined as the sum of all fixations and saccades within a given area of interest of infographics) was analyzed to check the visual attention to educational information. The raw sums of the score for the answers to the Subjective Numerical Literacy Scale, Subjective Graph Literacy Scale, and Subjective Linguistic Literacy Scale were analyzed to answer the question about the influence of processing information during debt education and the results of debt literacy education. The results of all literacy scales were calculated by using online scripts immediately after participants' submission of answers. The mean dwell time of visual attention was calculated from the eye tracking data for each area of interest (AOI) separately as well as for each infographic and in total using the SMI BeGaze 3.7 system. The area of interest (AOI) for each infographic was defined as the area of infographic with a margin from each side of 1.0° of visual angle in size.

### Statistics

The demographic properties of participants in the experimental and control group were compared with the use of a chi-square test (gender, education) and a *U*-Mann-Whitney test (age) for independent samples. The role of infographics in analyzing the change in debt literacy change was assessed using the W Wilcoxon signed ranks test for related samples, where the Debt Literacy Scale results from the first and second assessment were compared (measure 1 vs. measure 2) and the U Mann-Whitney test for independent samples (experimental vs. control). The role of infographics in analyzing debt literacy change was assessed with the use of a general linear model for repeated measures, where the Debt Literacy Scale results from the first and second assessment were a within-subjects factor (measure 1 vs. measure 2) and the group was a between-subjects factor (experimental vs. control). The same statistical procedure was used to assess changes in visual attention interaction between groups of debt literacy change (no increase vs. increase) and the infographic (first vs. second vs. third vs. fourth). To assess the changes in visual attention interaction between groups based on debt literacy change (no increase vs. increase) and the infographics (first vs. second vs. third vs. fourth) 54,980 fixations collected from 60 participants were applied. Mauchly's test of sphericity with epsilon correction was applied when necessary. Cohen's d, Hedges' g corrected for inequality of sample sizes, and partial η^2^ were used to assess the effect size of the results.

The comparison between the outcomes of the first and the second measurement of debt literacy was done with the use of a W Wilcoxon test for dependent samples. To verify whether variable A, that is, focusing on positives and negatives during debt education, in numerical literacy (variable C1), graph literacy (variable C2), or linguistic literacy (variable C3) moderates (variable B) the effectiveness of debt education, we used a mediation analysis procedure proposed by Hayes (Hayes, 2013) in model no 10. The full set of analyzed data with syntax in IBM SPSS® format can be found on the online repository at http://dx.doi.org/10.17632/f5s6cxk38f.1.

## Results

The experimental and control groups were comparable in gender composition [$\chi_{\left(1\right)}^{2}$ =0.51, *p* = 0.476, d = 0.138], age [z_U(108)_ = 0.179, *p* = 0.858, d = 0.034], and education level [$\chi_{\left(1\right)}^{2}$ = 1.67, *p* = 0.196, d = 0.251].

**Figure 3:**
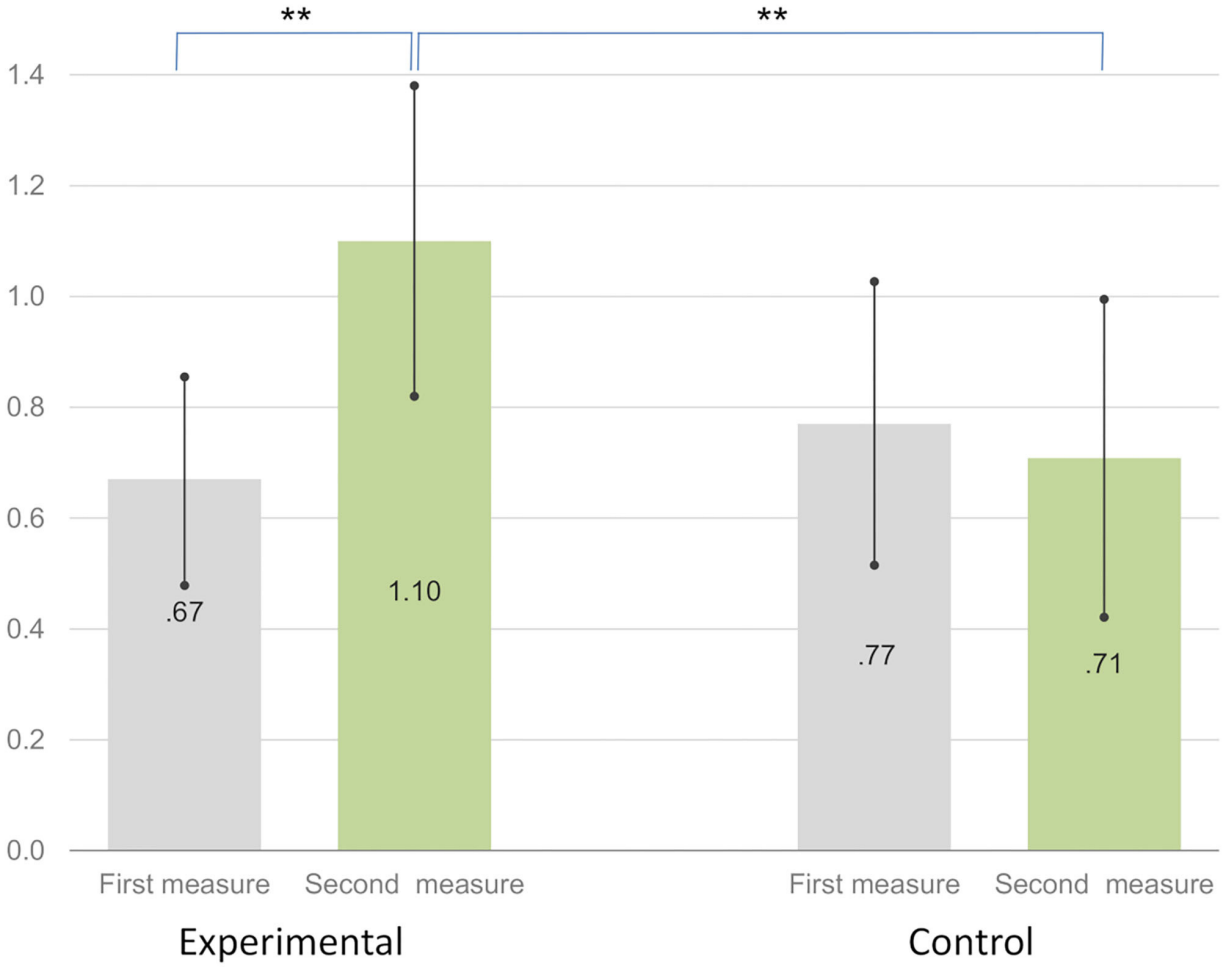
Debt literacy change in the experimental and control groups^ab^. ^a^Mean of debt literacy raw scores in the range 0–4. Significance: ^*^p < 0.05, ^**^p < 0.01, ^***^p < 0.001. ^b^Error bars represents 95.0% Lower and Upper CL for Mean.

The presence of educational debt information in infographics significantly improved the debt literacy score in the experimental group. The mean of the debt literacy score in the experimental group increased by 69% compared to the initial value—from 0.67 pt. (SD = 0.73) to 1.10 pt. (SD = 1.09). The effect of short-term debt education with the use of infographics in the experimental group was statistically significant [z_W(60)_ = 3.19, *p* = 0.001, d = 0.902]. The results are presented in Figure 3.

In the control group, debt literacy was 8.8% lower than the initial value, falling from 0.77 pt. (SD = 0.88) to 0.71 pt. (SD = 0.99). The difference between the experimental and control groups was significant in the second measure [z_U(108)_ = 2.162, *p* = 0.031, d = 0.425], but not significant in the first measure [z_U(108)_ = 0.435, *p* = 0.663, d = 0.084]. The result of presenting infographics with neutral information in the control group was not statistically significant [z_W(48)_ = 0.44, *p* = 0.663, d = 0.127].

To analyze the process and factors influencing potential debt education results in the experimental group, we extracted two subgroups of participants using the change in the debt literacy score: to the first group were classified those participants who increased their debt literacy (*N* = 21, M_DebtLiteracy−1_ = 0.48, SD_DebtLiteracy−1_ = 0.68; M_DebtLiteracy−2_ = 2.00, SD_DebtLiteracy−2_ = 1.00) and to the other group those who had no increase in debt literacy (*N* = 39, M_DebtLiteracy−1_ = 0.77, SD_DebtLiteracy−1_ = 0.74; M_DebtLiteracy−2_ = 0.62, SD_DebtLiteracy−2_ = 0.78).

**Figure 4:**
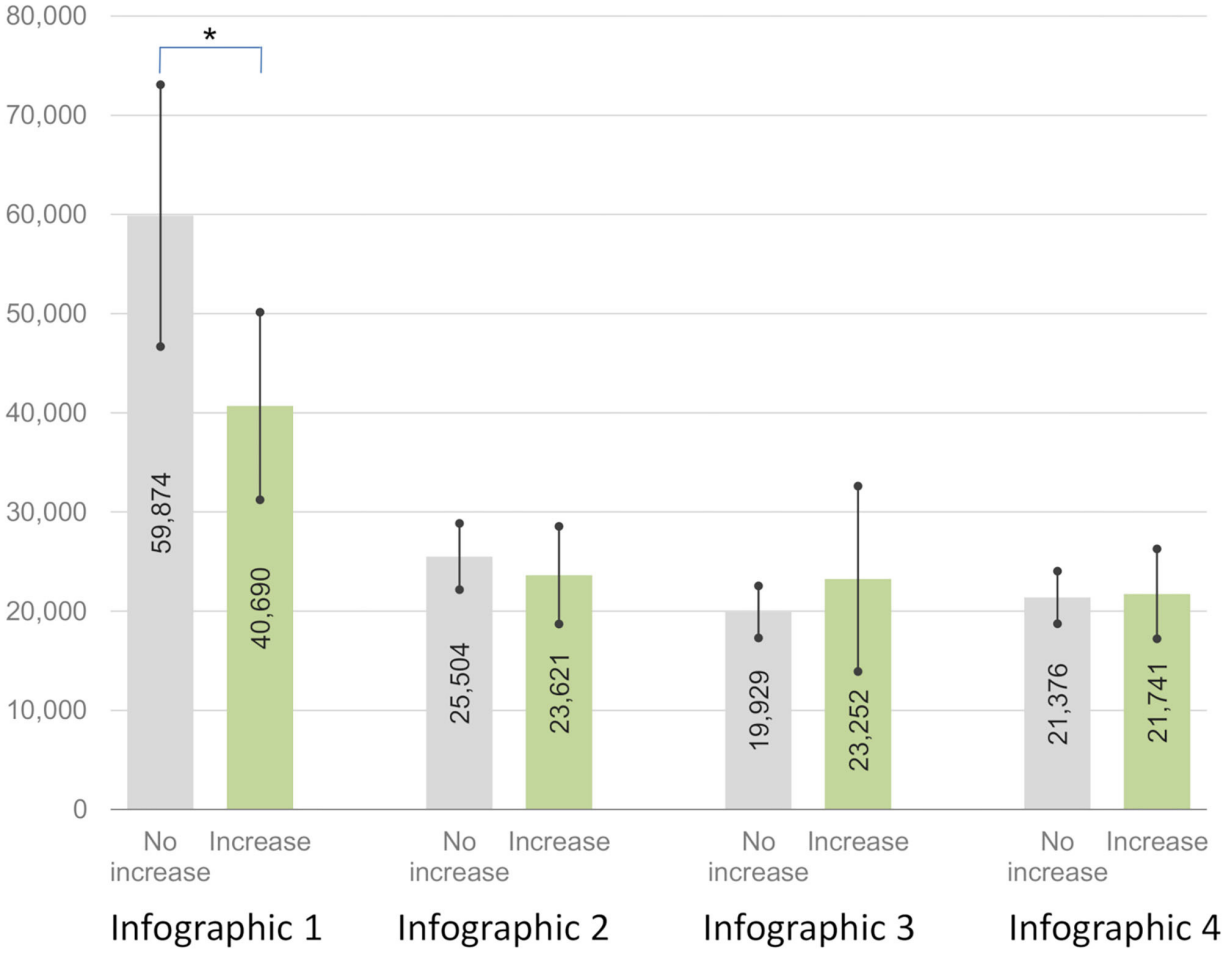
Dwell time of visual attention to infographics 1–4 for groups with no increase and increase in debt literacy as an effect of debt education^ab^. ^a^Mean dwell time in milliseconds. Significance: ^*^p < 0.05. ^b^Error bars represents 95.0% Lower and Upper CL for Mean.

There was a significant interaction between these two aforementioned groups in the experimental group (no increase vs. increase) and the mean dwell time of visual attention pertaining to the area of the presented infographics (first vs. second vs. third vs. fourth) [*F*_(1, 58)_ = 4.95, *p* = 0.030, $\eta_{\text{p}}^{2}$ = 0.079 ≈ d = 0.586]. The difference in the mean dwell time of visual attention pertaining to the area of the first presented infographic (Infographic 1) was significant, and lower-bound correction was applied [*t*_(58)_ = 2.02, *p* = 0.048, g = 0.546]. Participants from the group in which the debt literacy increased spent less time (M = 40689.78 ms, SD = 20757.57) processing information presented in the first infographic (Infographic 1) than participants from the other group (M = 59873.85 ms, SD = 40732.03). Differences between the mean dwell times pertaining to visual processing of other infographics were not significant [Infographic 2: *t*_(58)_ = 0.66, *p* = 0.510, g = 0.179; Infographic 3: *t*_(58)_ = 0.90, *p* = 0.375, g = 0.242; Infographic 4: *t*_(58)_ = 0.15, *p* = 0.879, g = 0.041]. Figure 4 presents the comparison.

The interaction between the two groups, which demonstrates the different effects of education on debt literacy in the experimental group (no increase vs. increase) and focus on the positives of debt shown in the infographics (first infographic vs. second vs. third vs. fourth) was significant, and lower-bound correction was applied [*F*_(1, 58)_ = 4.342, *p* = 0.042, $\eta_{\text{p}}^{2}$ = 0.070 ≈ d = 0.549]. Differences between the mean dwell time pertaining to the positives in the infographics were not significant [Infographic 1: *t*_(58)_ = 1.56, *p* = 0.124, g = 0.422; Infographic 2: *t*_(58)_ = 0.23, *p* = 0.822, g = 0.061; Infographic 3: *t*_(58)_ = 1.46, *p* = 0.154, g = 0.391; and Infographic 4: *t*_(58)_ = 0.97, *p* = 0.338, g = 0.262].

**Figure 5:**
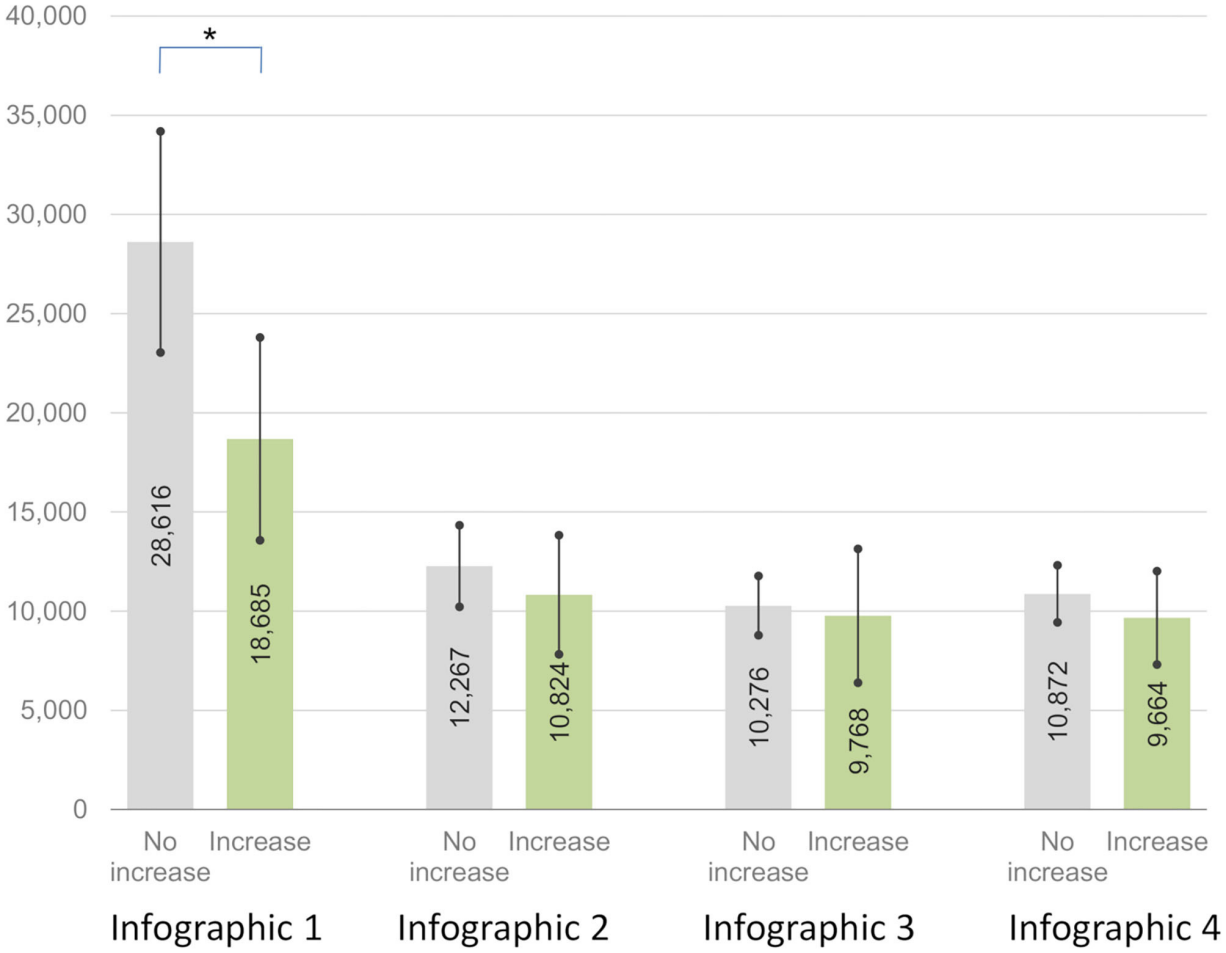
Dwell time of visual attention on negatives in infographics 1–4 for groups with no increase and increase in debt literacy as an effect of debt education^ab^. ^a^Mean dwell time in milliseconds. Significance: ^*^p < 0.05. ^b^Error bars represents 95.0% Lower and Upper CL for Mean.

The interaction between the two groups, which demonstrates the different effects of education on debt literacy in the experimental group (no increase vs. increase) and focus on negatives in infographics (first vs. second vs. third vs. fourth) was significant, and the lower-bound correction was applied [*F*_(1, 58)_ = 4.254, *p* = 0.044, $\eta_{\text{p}}^{2}$ = 0.068 ≈ d = 0.540]. The differences between the mean dwell time pertaining to negatives in Infographic 1 were significant, and for the remaining infographics, it was insignificant [Infographic 1: *t*_(58)_ = 2.38, *p* = 0.021, g = 0.645; Infographic 2: *t*_(58)_ = 0.83, *p* = 0.411, g = 0.224; Infographic 3: *t*_(58)_ = 0.33, *p* = 0.744, g = 0.089; and Infographic 4: *t*_(58)_ = 0.95, *p* = 0.346, g = 0.257]. Figure 5 presents the comparison.

Table 1 presents the differences in the three aspects of assessed information literacy between the two groups—those who increased their level of debt literacy, and those who did not—of participants. The results revealed no significant differences between groups in any of: numerical literacy [*t*_(58)_ = 1.26, *p* = 0.213, g = 0.333], graph literacy [*t*_(58)_ = 0.46, *p* = 0.650, g = 0.127], or linguistic literacy [*t*_(58)_ = 0.43, *p* = 0.666, g = 0.110].

**Table 1:** Differences in numeracy, graph literacy, and linguistic literacy between groups with no increase in literacy and those with an increase because of debt education.

**Literacy**	**Group**	***N***	**Mean**^**a**^	**Std deviation**	***t***^**b**^	***p***	**g**
Numerical	No increase	39	30.00	7.81	−1.26	0.213	0.333
	Increase	21	32.67	7.84			
Graphical	No increase	39	21.87	5.93	0.46	0.650	0.127
	Increase	21	21.19	4.64			
Linguistic	No increase	39	22.21	4.11	0.43	0.666	0.110
	Increase	21	21.76	3.05			

a *Range of mean of raw scores: Information 18–108; Numerical 8–48; Graphical 5–30; Linguistic 5–30*.

b Significance: ^*^p < 0.05 ^**^p < 0.01 ^***^p < 0.001.

**Figure 6:**
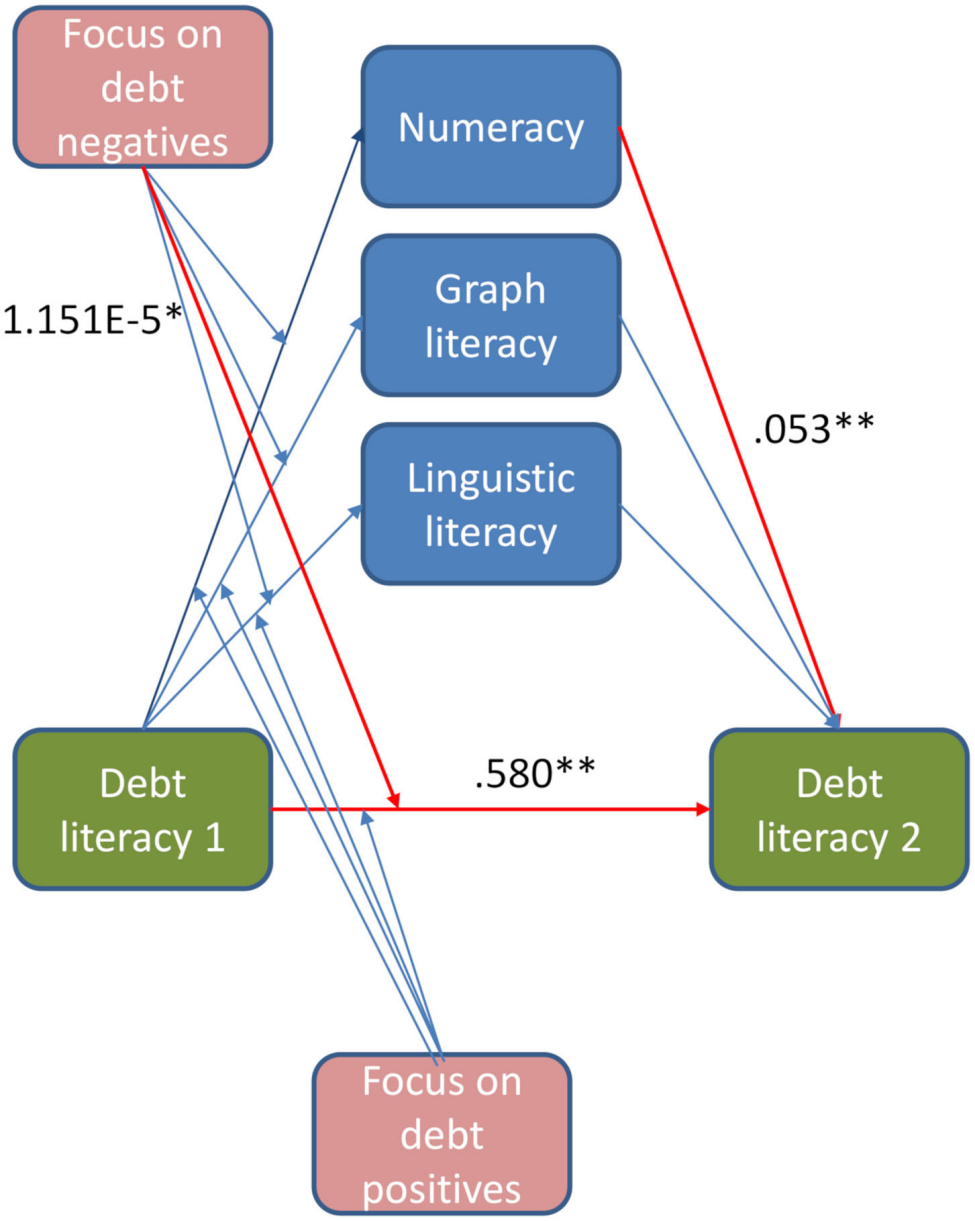
The role of visual attention focus on debt positives, debt negatives, numeracy, graph literacy, and linguistic literacy in debt education^ab^. ^a^Unstandardized beta coefficient weights from the mediation test. ^b^Significance: ^*^p < 0.05, ^**^p < 0.01.

The unstandardized weights of the beta coefficient from the mediation test are shown in Figure 6. The overall model explains 35.5% of the variability in debt education outcomes [*F*_(8, 51)_ = 3.50, *p* = 0.003, R^2^ = 0.355]. The initial level of debt literacy is the most significant predictor of debt education results with the use of infographics [*t*_(51)_ = 3.16, *p* = 0.003]. The higher the initial debt literacy, the higher the final debt literacy, and the lower the debt education increase.

Numeracy mediates the relationship between the initial and final debt literacy, thereby strengthening the results of debt education [*t*_(51)_ = 2.87, *p* = 0.006]. Visual attention on the negatives of debt in infographics moderates the process of debt education reducing outcomes [*t*_(51)_ = 2.17, *p* = 0.035]; however, the interaction between initial debt literacy and focus on negatives is not significant [*t*_(51)_ = 1.56, *p* = 0.126]. Graph and linguistic literacy, as well as the focus on positives in infographics, are insignificant as mediators and moderators [accordingly: *t*_(51)_ = 1.52, *p* = 0.135; *t*_(51)_ = 0.40, *p* = 694, and *t*_(51)_ = 1.51, *p* = 0.138].

## Discussion

### Efficiency of Infographics-Based Debt Education

This study provides evidence that infographics-based education may be effective, at least in the short run. The scale of debt literacy growth of 2/3 of the entry level in the experimental group after exposition to a series of 4 simple infographics is very promising, suggesting the high potential of using infographics in debt education. It is likely that even such a short-term infographics-driven intervention, leading to a significant though small increase in debt literacy, may be relevant in educational programs supporting healthy financial behavior. For instance, infographics-based educational material similar to the one designed in our study could be shared immediately before the credit decision (i.e., credit/loan selection), as a part of the creditworthiness evaluation process, or as a part of the contents displayed on the website that allows various credit offers to be compared. Additionally, the advantage of such an educational intervention is its relatively low cost, which comes down mainly to the design and production of the infographics.

The effect size of short-term debt education results is satisfying, but the final level of debt competencies measured after the micro scale of the experimental process is still low. Our study showed a low level of debt literacy among the participating individuals. To a large extent, the participants lacked the knowledge and skills deemed useful in typical debt-related situations. These results are consistent with the findings of previous studies (Disney and Gathergood, 2011; Sole, 2014; Lusardi and Tufano, 2015; van Ooijen and van Rooij, 2016; Cwynar et al., 2018b). This raises warranted concerns because debt literacy shortcomings may hinder professional activity and entrepreneurship (Lusardi, 2015; Klapper et al., 2018), and cause difficulties in everyday financial management (Klapper et al., 2013). One possible reason for the results lies in the limits of computer education procedures. Lack of adaptation to the audience, their abilities, skills, knowledge and experience, makes good educational results hard to achieve. To tackle the problem we should offer education that is sensitive to participant characteristics.

This finding also points to the general methodological problem of strategies and instruments used for evaluating financial education results when the survey questions are not exactly comparable to the content of educational materials; this potentially lowers the outcomes (Robinson et al., 2016). We assume that the problem should not be studied only in terms of potential inconsistency between the education topics and the measure tool content, but also in terms of verifying the potential positive transfer between awareness of the debt positives and negatives and attentiveness during simulated and real debt decisions. The infographics used in our experiment focused on participants' attention on key debt awareness topics; further, they encouraged them to understand the general rules that are useful for safely and effectively managing their loans through examples appealing to their critical thinking. Noticing the significant results of this procedure in the presented experiment, we may assume positive mobilization of participants' attentiveness to the Debt Literacy Test details, where simple but thoughtful analysis is expected (Lusardi and Tufano, 2015). The content of the infographics should, therefore, be studied in future research to verify the role of motivation and its significance for the attentiveness developed by the debt education process.

The experiment was not directly focused on verification of the relation between top-down visual attention focus and bottom-up verbal interpretation process (Wolfe et al., 2003). The final level of debt literacy was linked to participants' learning about the structure of infographics. Those who focused attention on relevant information proved good performance. The results suggest a staging of bottom-up processes, triggered in the first phase of infographic analysis, when top-down processes were triggered after learning the rules for conveying information in infographics. However, the experiment does not settle the issue of staging. Rather, it provides grounds for further exploration. The results do not provide any clear argument for the discussion on learning styles. They only allow us to assume that mental operations during learning from infographics moderated by top-down process can be sensitive to individual characteristics of the learning person, especially those connected with the preference for numerical data and with focus on positives of debt (Hoffmann and McNair, 2019).

Another significant area of focus for future studies is the long-term verification of debt education results we proved by taking a very short-term example. Studies show debatable and mixed long-term results of general economic education and the correlates of this process (Fernandes et al., 2014). Brown et al. (2016) confirmed the positive effect of general mathematics-related cognitive skills improvements on the increase of incomes and savings with unchanged debt and repayment difficulties; however, we did not analyze the long-term debt education outcomes.

### The Mechanisms of Infographics-Based Education

To examine the mechanisms underlying debt literacy increments, we carried out a comparative analysis of the two participating groups: those who exhibited an increased debt literacy level (increase group) and those who did not (no increase group). These groups were compared in terms of visual attention to infographics and the components of information literacy selected: numerical, graph, and linguistic.

### Processing of Visual Information

We found that the increase group focused visual attention on educational material for a shorter time than the no-increase group—an effect that provided the basis for the rejection of H1. The differences in the mean dwell time were quite considerable (~20%) and occurred almost exclusively when the participants were exposed to the first infographic. The three remaining infographics attracted the visual attention of both groups for comparable lengths of time. This result is interpreted as not being contradictory to findings proving the need for attention to be paid to the processed information for better learning (Bosse and Valdois, 2009), problem solving (Madsen et al., 2012; Wegbreit et al., 2012), or the probability of buying (Grebitus et al., 2015; Rihn and Yue, 2016) that are taken as H1 assumptions. The result we obtained can be analyzed in the context of selective visual processing of complex information. The ability to select information significant for decisions about future behavior is one of the most important for effectively solving daily life tasks and avoiding attention depletion (Moore and Zirnsak, 2017). The information that was processed by the participants in our experiment was complex and consisted of two parts. The debt information was different in each infographic, whereas the general structure of each infographic was the same, that is, the textual information was always presented above the graphical information. The structure of stimuli containing the infographic text on its right and left sides and different graphics on both sides was visually analyzed by participants when the first infographic was presented. When the structure of infographics was recognized and the participants understood that the text was the same on both sides of the infographics, they paid less attention to the rest of the infographics without losing significant educational information. This interpretation supports the observed tendency to give more attention to messages not seen by participants before (Chua et al., 2018).

Our results may also suggest that the increase group displays higher visual cognitive abilities, allowing for faster recognition of what lies behind the differences in stimuli layout (Assel et al., 2003; Green, 2010). In other words, the results may suggest that our increase group learned faster. The similar mean dwell time found for the other infographics may be indicative of the fact that the no increase group also learned the “informational pattern” reflected in the layout of the infographics, although later than—not so fast as—the increase group.

We found that numeracy mediates the relationship between the initial and final debt literacy, strengthening the results of debt education. In this respect, our findings are similar to those obtained by Grohmann et al. (2015), who showed that schooling indirectly influences financial literacy by increasing numeracy. We also found that visual attention that focused on negatives of debt in infographics moderates the process through which the effect of debt education is hampered. The role of numeracy is well established in the relevant literature, and our study confirms the significance of numerical education for appropriate debt decisions (Lusardi, 2012; Grotlüschen et al., 2019; Hoffmann and McNair, 2019). The role of focusing on debt negatives was derived from other studies; however, it has not yet been confirmed (Cloninger, 1986; Tversky and Kahneman, 1992; Gross, 2006; Cubillo et al., 2019). Assuming the positive role of debt education, the infographics should focus on the positives to empower debt literacy.

Theoretical assumptions of the experiment were confirmed. The top-down model of visual attention applied in the presented experiment assumes the existence of a regulatory factor that focuses on the selected aspects of perceived information. Such intentional regulation was evident in focusing attention on infographics interpreted as significant for participants. It has been confirmed that general values can play such a regulatory role for visual attention (Anderson and Halpern, 2017), as well as consumers' interests (Gidlöf et al., 2017). However, it was found that not all values influence visual attention and behavior. For example, concerns regarding environmental issues do not promote the purchase of certified forest coffee, whereas illustrations of forests on certified forest coffee labels attract participants' visual attention and further stimulate actual purchases of certified forest coffee by as much as 22 percentage points for each second of attention (Takahashi et al., 2018). Thus, the value- or interest-driven attentional paradigm is worth considering in future studies to verify the possibility of utilizing specific topics and forms of infographics-based debt education to enhance educational results. To study the role of the content or form of infographics in grabbing participants' visual attention because of their values or interests, the random order and simplified content of infographics should be applied in a paradigm different from that applied in the current study.

### Cognitive Determinants of Infographics-Based Education

We found that the two groups—the increase group and the no increase group—compared in this study did not differ significantly in their information literacy, a finding that allowed us to reject H2. This result indicates that the process of debt literacy acquisition need not necessarily be leveraged by the factors comprising our measure of information literacy. This result can support doubts about the relationship between numeracy and economic literacy (Gustman et al., 2012). Specifically, this finding means that although our debt literacy test was largely numerical (questions that required dealing with numbers, conducting computations, etc.), it turned out that debt literacy may be improved exclusively by exposure to infographics (an image plus a short text), without accompanying numeracy support. This is important because previous studies reported a significant relationship between financial literacy and numeracy (Banks et al., 2011; Cole et al., 2011; Sole, 2014; Donleavy et al., 2018). However, the role of numerical, graph, and linguistic literacy in debt literacy education should be verified by using other test formats to measure the studied literacies; this can provide additional support to our findings.

Overall, this study provides insight into both methodological and educational issues related to the use of infographics in debt education. The results confirm the purposeful use of infographics in short-term debt education. The infographics-based debt education results can probably be enhanced by the originality of the presented information and a longer and broader education process; further, expanding the scope of competencies tested by the Debt Literacy Scale can remove the ceiling effect noticed in the analysis.

### Limitations

Like most experiments, ours was also a small scale one. The study is not generalizable to a broad population, retaining validity primarily for a group of ~30 years of age (18–60), with a high school or college education, and predominantly female. Future research should verify our findings both in a larger study sample and over a longer period of time. Overall, there is very little research dedicated to different formats of financial education. Therefore more studies are needed to test different formats, particularly those most promising in terms of online opportunities.

In our study, we did not analyze the role of learning styles in the effectiveness of the tested infographics. Nonetheless, the mediating influence of numeracy can be inferred also from this theory. Future research could consider this factor by examining how individuals with different learning styles respond to the educational material we designed.

In our study, we focused on debt literacy. While we surmise that domain-specific content will not make much difference to the effectiveness of using infographics in financial education as such, scientific integrity dictates that we examine how they perform with respect to other aspects of financial literacy—e.g., investment literacy, insurance literacy, etc.—as well as with respect to financial literacy in general.

### Implications, Recommendations and Directions for Future Research

Our findings have implications for both the theory and practice of financial education, and for both the academic world and for policy- and decision-makers. Social learning theory (Bandura, 1977) posits that learning is a social process. Generally, individuals like to interact, including when they are learning. Today, many working adults do not have enough time to educate themselves financially in the traditional way by attending in-class workshops. Online education can be an attractive alternative for them. However, we should always pay attention to individualization of such education, taking into account participant characteristics which moderate learning outcomes. This does not at all have to entail losing the benefits of the social levers of learning. It is only necessary to design an educational program so that it assumes a learning community. It is quite presumable that in the era of the enormous popularity of social media, when social needs are more and more frequently met online, many people will benefit from such a form of education, especially persons with disabilities experiencing a lack of social, cultural, and economic power (Kattari, 2015).

In our experiment, we showed that financial infographics, which are perfect for online education, increase debt literacy. We confirmed the supporting role of numerical competencies in the process. One new interesting result of the current study is that focus on negatives of debt is detrimental to the results of debt education. Focus on negatives can be attributed to risk-aversion as well as to negative attitudes toward borrowing, developed during economic education. Future studies may examine whether social factors (i.e., those responsible for the benefits of learning in a learning community) or support for rational attitudes toward debt can amplify the positive effects of such programs. This could shed a new light on this very timely aspect of social learning theory.

We also showed that laboratory experiments—including those using innovative measurement techniques like eye tracking—are a useful method for evaluating the outcomes of financial education. The effectiveness of micro-education could be a predictor for general education. The methodological paradigm of studies on micro-education using eye trackers can be applied to investigate educational outcomes in any subject. It is worth considering the verification of different stimuli properties, like the amount of text, the shape and color of the objects and the background (Ceravolo et al., 2019).

In the literature review section we pointed out that inconclusive results of research on the effectiveness of financial education may be due to ineffective program evaluation. Laboratory experiments can avoid the controversy associated with causal inference, which often accompanies studies of the effectiveness of financial education (Fernandes et al., 2014). Policy- and decision-makers should design financial education interventions with this important finding in mind. Researchers, in turn, should note that lab experiments are a very useful tool for establishing causal inference in financial literacy studies.

Policy- and decision-makers, as well as practitioners, can also benefit from the results of our study in that it demonstrates the role of educational format in achieving training outcomes. Although we did not compare the effectiveness of infographics to other formats, our results support the anecdotal evidence that custom (non-traditional) formats using pictorials, ideally suited for online use, can be effective in financial education. Given that financial education to date has focused primarily on content and less on form, our findings may be useful for those who design financial education interventions and implement educational programs.

From a purely practical standpoint, our results can be taken as a very clear indication of how infographic educational material should be designed. Our educational material has a user-friendly format, is interactive, reusable, and closely linked to key aspects of the annual percentage yield (APY), a particularly important parameter for understanding the implications of using different loan products.

## Data Availability Statement

The raw data supporting the conclusions of this article will be made available by the authors, without undue reservation.

## Ethics Statement

The studies involving human participants were reviewed and approved by The University of Economics and Innovation Ethics Committee. The patients/participants provided their written informed consent to participate in this study.

## Author Contributions

RP: design of the work, data collection, data analysis and interpretation, and drafting the article. AC: conception, data interpretation, drafting the article, and critical revision of the article. WC: critical revision of the article and final approval of the version to be published. All authors contributed to the article and approved the submitted version.

## Conflict of Interest

The authors declare that the research was conducted in the absence of any commercial or financial relationships that could be construed as a potential conflict of interest.
